# In vivo therapy of osteosarcoma using anion transporters-based supramolecular drugs

**DOI:** 10.1186/s12951-023-02270-x

**Published:** 2024-01-13

**Authors:** Zeyu Zheng, Xiaoan Wei, Yangyang Lin, Siyue Tao, Hui Li, Zhongyin Ji, Hongxin Wei, Jiayan Jin, Fengdong Zhao, Chao Lang, Junhui Liu, Jian Chen

**Affiliations:** 1 Department of Orthopaedic Surgery, Sir Run Run Shaw Hospital, Zhejiang University School of Medicine, Hangzhou, China; 2 Key Laboratory of Musculoskeletal System Degeneration and Regeneration Translational Research of Zhejiang Province, Hangzhou, China; 3 South China Advanced Institute for Soft Matter Science and Technology, School of Emergent Soft Matter, South China University of Technology, Guangzhou, 510640 China; 4 Guangdong Provincial Key Laboratory of Functional and Intelligent Hybrid Materials and Devices, South China University of Technology, Guangzhou, 510640 China

**Keywords:** Osteosarcoma, Anion transporter, Osteosarcoma targeting peptide, Liposomes, Tumor targeting, Regulate tumor immune microenvironment

## Abstract

**Background:**

Osteosarcoma represents a serious clinical challenge due to its widespread genomic alterations, tendency for drug resistance and distant metastasis. New treatment methods are urgently needed to address those treatment difficulties in osteosarcoma to improve patient prognoses. In recent years, small-molecule based anion transporter have emerged as innovative and promising therapeutic compound with various biomedical applications. However, due to a lack of efficient delivery methods, using ion transporters as therapeutic drugs in vivo remains a major challenge.

**Result:**

Herein, we developed self-assembled supramolecular drugs based on small-molecule anion transporters, which exhibited potent therapeutic effect towards osteosarcoma both in vitro and in vivo. The anion transporters can disrupt intracellular ion homeostasis, inhibit proliferation, migration, epithelial-mesenchymal transition process, and lead to osteosarcoma cell death. RNA sequencing, western blot and flow cytometry indicated reprogramming of HOS cells and induced cell death through multiple pathways. These pathways included activation of endoplasmic reticulum stress, autophagy, apoptosis and cell cycle arrest, which avoided the development of drug resistance in osteosarcoma cells. Functionalized with osteosarcoma targeting peptide, the assembled supramolecular drug showed excellent targeted anticancer therapy against subcutaneous xenograft tumor and lung metastasis models. Besides good tumor targeting capability and anti-drug resistance, the efficacy of the assembly was also attributed to its ability to regulate the tumor immune microenvironment in vivo.

**Conclusions:**

In summary, we have demonstrated for the first time that small-molecule anion transporters are capable of killing osteosarcoma cells through multiple pathways. The assemblies, OTP-BP-L, show excellent targeting and therapeutic effect towards osteosarcoma tumors. Furthermore, the supramolecular drug shows a strong ability to regulate the tumor immune microenvironment in vivo. This work not only demonstrated the biomedical value of small-molecule anion transporters in vivo, but also provided an innovative approach for the treatment of osteosarcoma.

**Supplementary Information:**

The online version contains supplementary material available at 10.1186/s12951-023-02270-x.

## Background

Osteosarcoma, also known as osteogenic sarcoma, is the most prevalent malignant tumor of bone. Derived from the mesenchymal cells, osteosarcoma is one of the most lethal and malignant tumors in children and adolescents [1–3]. Currently, the main clinical treatment for osteosarcoma is neoadjuvant chemotherapy (high-dose methotrexate, doxorubicin, and cisplatin) combined with local surgical resection [4]. However, due to the high incidence of drug resistance and distant metastasis, the survival period of patients remains short [5]. Additionally, because osteosarcoma has widespread genomic alterations, it is difficult to identify specific molecular targets for effective precise therapy, which often leads to adverse effects during chemotherapy [6]. Therefore, new treatment methods are urgently needed to address drug targeting, drug resistance, and distant metastasis in osteosarcoma to improve patient prognoses.

Intracellular ion stability is crucial for maintaining cell osmotic pressure and proper physiological functions, whereas imbalances in intracellular ion homeostasis will lead to cell death. Studies have shown that drastic changes in intracellular ion concentrations, such as Cl^−^, Ca^2+^, and K^+^, can lead to cell death through apoptosis, autophagy, necroptosis, ferroptosis, oxidative stress, and endoplasmic reticulum (ER) stress [7–11]. In this context, various synthetic structures have been proposed to mimic the physiological transport function in living systems [12–20]. In particular, drug-like small-molecule ion transporters [21–26] have been proposed as a promising cancer therapy by facilitating anion transport into cells, which could induce apoptosis through caspase-dependent pathways [27–29]. In cells, anion transporters have been shown to facilitate Cl^−^ and Na^+^ influx into cytosols, which will lead to reactive oxygen species (ROS) increase, release of cytochrome c from mitochondria, and finally apoptosis through caspase-dependent pathways [27]. Squaramide-based ion transporter has also been shown to disrupt autophagy [29], which renders it a promising candidate as cancer therapy. However, small-molecule based anion transporters lack targeting abilities, which limits their curing effect in vivo. Previous studies have thus been limited to in vitro experiments. The in vivo therapeutic effect of anion transporters for cancers thus needs further investigation.

Furthermore, because osteosarcoma lacks specific surface markers, the development of targeting peptides for osteosarcoma has become a new research hotspot. For instance, Liu et al. developed intranuclear nanoribbons formed upon dephosphorylation of leucine-rich L- or D-phosphopeptide catalyzed by alkaline phosphatase (ALP) to selectively kill osteosarcoma cells. The peptide can directly kill osteosarcoma cells and minimize the drug resistance caused by repeated treatment [30]. Additionally, Lin et al. screened peptides that can target osteosarcoma cells through phage display techniques. The peptide only has the ability to recognize osteosarcoma cells but can’t kill osteosarcoma cells by itself. By self-assembling with nanodrugs, it can selectively kill osteosarcoma cells in vivo [31]. Furthermore, reprogramming tumor-associated macrophages (TAMs) from the M2 phenotype that inhibits tumor immunity to the M1 phenotype that promotes tumor immunity is another important target for tumor treatment and many drugs are designed to regulate the tumor immune microenvironment as a means of treating tumors [32, 33].

In this study, we selected ion transporter PTU, TFPTU, and BTFPTU that can self-integrate into cell membranes as candidate compounds. Through in vitro experiments, we identified BTFPTU as having the best tumor killing effect. Osteosarcoma-targeting peptides (OTP) and BTFPTU were loaded into liposomes through self-assembly process for osteosarcoma treatment. Through in vitro and in vivo experiments, we found that the assembled supramolecular drug (OTP-BP-L) had good targeting and killing effects on osteosarcoma cells, as well as anti-drug resistance and the ability to regulate the tumor immune microenvironment. These results demonstrate that OTP-BTFPTU can be used as a new strategy for osteosarcoma treatment.

## Result

### Synthetic procedures and characterization data of PTU, TFPTU and BTFPTU

**Fig. 1:**
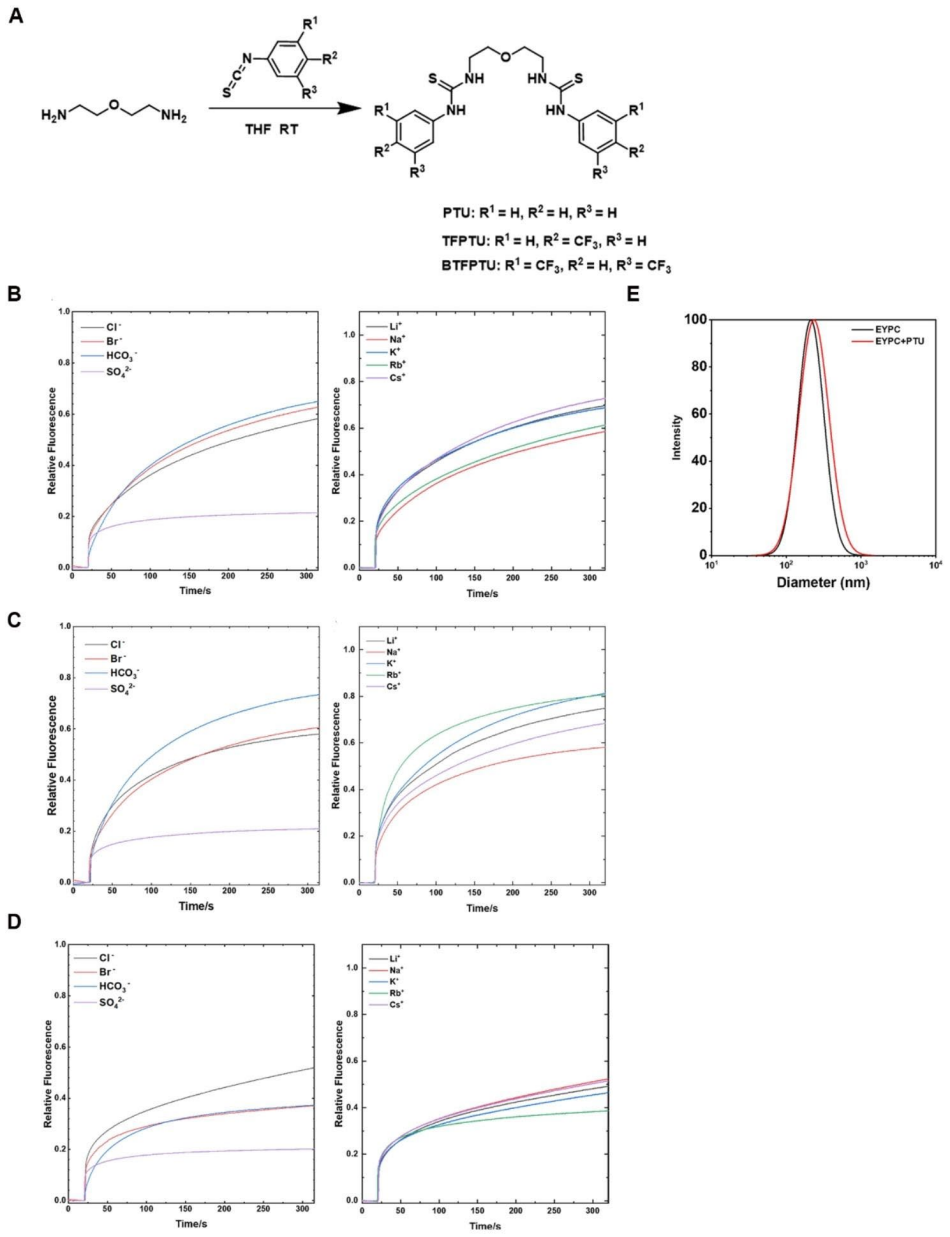
Synthetic procedures and transmembrane ion transport properties of PTU, TFPTU and BTFPTU. (**A**) Synthetic routes of PTU, TFPTU and BTFPTU. (**B–D**) Anionic and cationic selectivity of PTU, TFPTU and BTFPTU determined using the HPTS assays. (**E**) Dynamic light scattering (DLS) measurements of large unilamellar vesicles (LUVs) made from blank EYPC and EYPC with anion transporter PTU

Bimodal ion transporters used in this study were based on thiourea groups, and were synthesized by reacting bis(2-aminoethyl) ether with corresponding isothiocyanates (Fig. 1A). Detailed synthetic processes have been reported somewhere else [34] and the ^1^H NMR spectrum of PTU, TFPTU and BTFPTU in DMSO-*d*6 were shown in the Fig. S1A-C.

We tested the cationic and anionic selectivity of PTU, TFPTU and BTFPTU by using 8-hydroxypyrene-1,3,6-trisulfonic acid (HPTS) assay. The results showed that the ion transporters exhibited distinct ion selectivity towards, and similar transporting activities for cations (Fig. 1B-D). The diameters of LUVs of egg yolk phosphatidylcholine (EYPC) and EYPC with PTU were similar, indicating that insertion of ion transporters has minimum effect on lipid membranes (Fig. 1E).

### BTFPTU inhibits the proliferation and migration of osteosarcoma cells in vitro

**Fig. 2:**
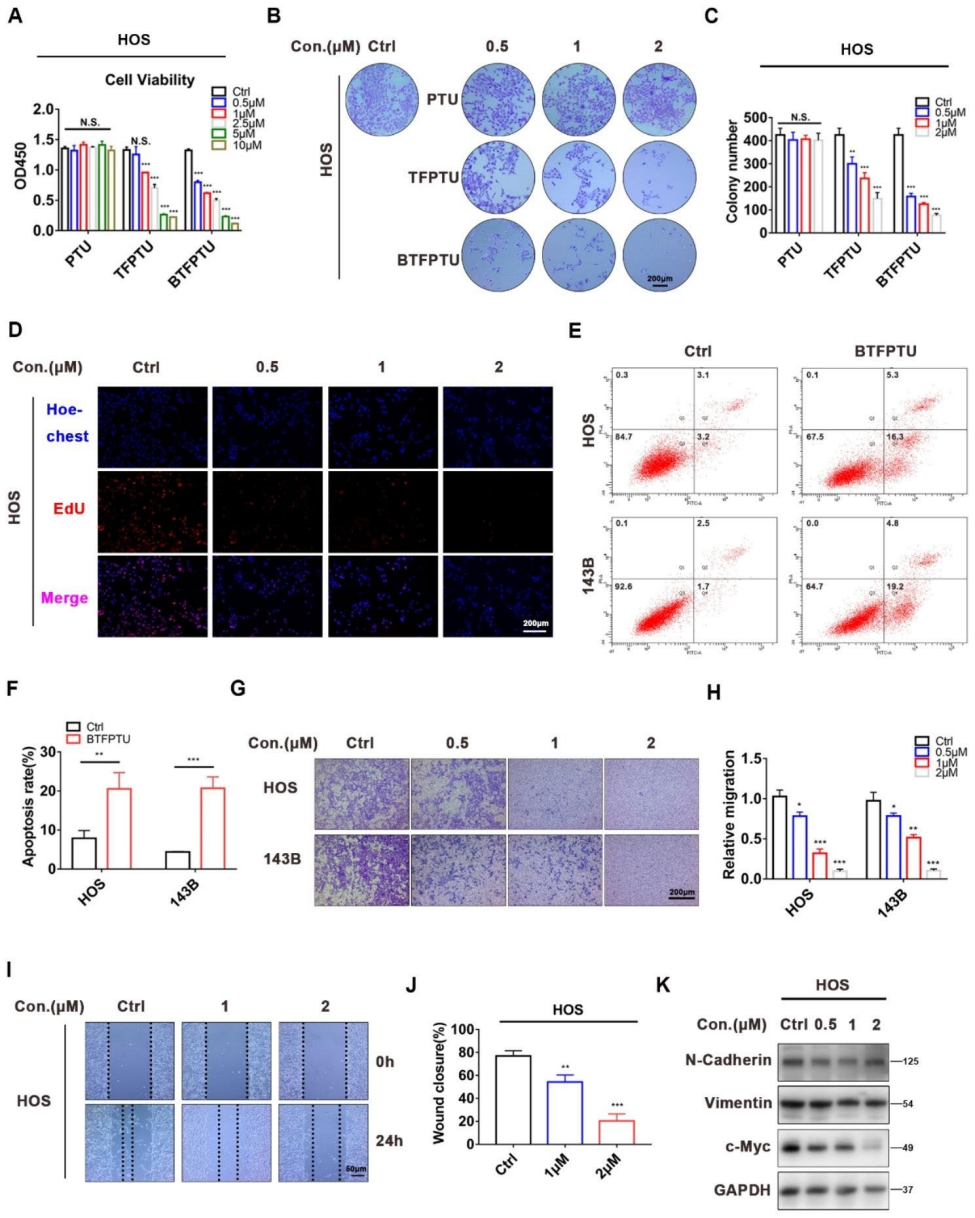
BTFPTU inhibits the proliferation and migration of osteosarcoma cells in vitro. (**A**) Viability of HOS cells with PTU, TFPTU or BTFPTU treatment for 24 h using Cell Counting Kit-8 assay. (**B**) Effect of PTU, TFPTU or BTFPTU treatment for 48 h on the colony-forming ability of HOS cells based on colony formation assays. Scale bar, 200 μm. (**C**) Quantification of the colony number. (**D**) HOS cells were treated with different concentrations of BTFPTU for 24 h, and the cell proliferation was characterized by the EdU assay. Scale bar, 200 μm. (**E**) Flow cytometry to determine apoptosis of HOS and 143B cells with or without BTFPTU (2 µM) treatment for 12 h by Annexin V-FITC/PI apoptosis detection. (**F**) Quantification of apoptosis rate of HOS and 143B cells. (**G**) Cell migration abilities of HOS and 143B cells treated with different concentrations of BTFPTU for 24 h were measured by Transwell migration assays. Scale bar, 200 μm. (**H**) Quantification of the migration abilities of HOS and 143B cells. (**I**) The wound healing assay was used to evaluate the migration abilities of HOS cells treated with different concentrations of BTFPTU for 24 h. Scale bar, 50 μm. (**J**) Quantification of the wound closure rate. (**K**) The protein expression of N-Cadherin, Vimentin and c-Myc were measured by Western blot analysis in HOS cells treated with varying concentrations of BTFPTU for 24 h. The data represent the mean ± SD of three independent experiments. *p < 0.05, **p < 0.01, ***p < 0.001 for a comparison with the control group or as indicated

Studies have shown that disruption of intracellular ion concentration homeostasis can activate multiple pathways such as apoptosis, autophagy, pyroptosis, ferroptosis, oxidative stress, and ER stress, ultimately leading to cell death [7–11]. Based on the above theory, we investigated whether PTU, TFPTU, and BTFPTU could disrupt the ion homeostasis of osteosarcoma cells and kill them. Firstly, osteosarcoma cell lines (HOS and 143B cells) were treated with increased concentrations of PTU, TFPTU, and BTFPTU. CCK-8 assay showed that PTU had minimum effect on cell viability, while TFPTU and BTFPTU exhibited concentration-dependent cytotoxicity against osteosarcoma cells and BTFPTU had stronger cell toxicity than TFPTU (Fig. 2A and Fig. S2A). Similarly, clone formation assay showed that TFPTU and BTFPTU concentration-dependently impaired colony-forming ability of osteosarcoma cells (Fig. 2B, C and Fig. S2B, C). Based on the results of CCK-8 and clone formation assays, we selected BTFPTU, which exhibited the strongest cytotoxicity against osteosarcoma cells, for following experiments.

EdU assay further confirmed that BTFPTU reduced cell viability (Fig. 2D and Fig. S2D), and flow cytometry showed that BTFPTU significantly increased the apoptosis of osteosarcoma cells (Fig. 2E, F). After confirming that BTFPTU could significantly inhibit the proliferation and promote apoptosis of osteosarcoma cells, we further explored whether it will affect the migration ability of osteosarcoma cells. As revealed by Transwell and wound healing assays (Fig. 2G-J and Fig. S2E, F), the migration ability of osteosarcoma cells was impeded after treatment with BTFPTU. The epithelial-mesenchymal transition (EMT) process and unlimited proliferation ability are important prerequisites for the migration and distant metastasis of osteosarcoma cells [35]. The EMT markers N-cadherin, vimentin and proto-oncogene c-Myc in osteosarcoma cells were significantly downregulated with BTFPTU treatment (Fig. 2K and Fig. S2G, H). In summary, BTFPTU can significantly inhibit the proliferation and migration ability of osteosarcoma cells in vitro.

### BTFPTU triggers transcriptional reprogramming of HOS cells

**Fig. 3:**
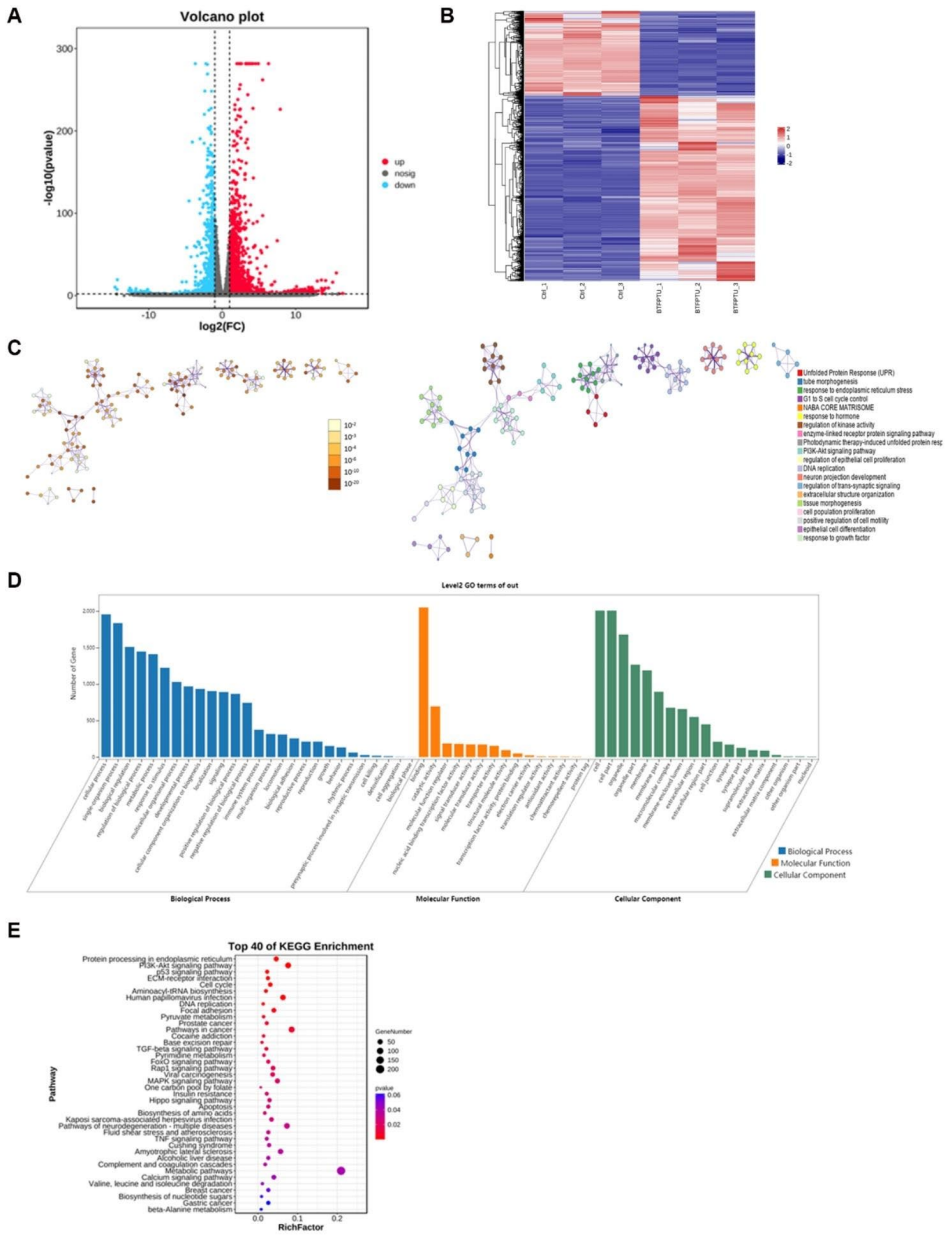
BTFPTU triggers transcriptional reprogramming of HOS cells. (**A**) Volcano plot of RNA sequencing (RNA-seq) analysis on HOS cells treated with either PBS or BTFPTU for 24 h. (**B**) The differential gene clustering heatmap of RNA-seq analysis. (**C**) The network of enriched terms of RNA-seq analysis. The left figure was colored by P-value and terms containing more genes tended to have a more significant P-value. The right figure was colored by cluster ID and nodes share the same cluster were typically close to each other. (**D**) KEGG enrichment of RNA-seq analysis. (**E**) GO enrichment of RNA-seq analysis. p < 0.05 is defined as having a significant difference for comparison with the control group

In order to further investigate the mechanism of osteosarcoma cell death induced by BTFPTU, we performed RNA sequencing (RNA-seq) analysis on HOS cells treated with either PBS or BTFPTU for 24 h (Fig. 3). The volcano plot showed that, compared with the control group, there were 2218 upregulated genes and 994 downregulated genes with statistically significant differences in expression level in the BTFPTU treatment group (Fig. 3A). The results of the differential gene clustering heatmap showed that control and BTFPTU treatment group could be well clustered, indicating that the gene expression characteristics in each group were similar (Fig. 3B). Furthermore, the network of enriched terms showed that the BTFPTU treatment caused prominent changes in the expression of genes related to cell proliferation and death, such as unfolded protein response (UPR), response to ER stress, G1 to S cell cycle control, PI3K-Akt pathway, and cell population proliferation (Fig. 3C). In KEGG (Kyoto encyclopedia of genes and genomes) enrichment analysis, PI3K-Akt, P53, TGF-β, MAPK pathway, cell cycle, DNA replication and apoptosis pathway all entered the top 40 (Fig. 3D). GO (Gene Ontology) enrichment analysis showed that BTFPTU altered pathways of biological regulation, protein binding, and cell membrane component, which we were particularly interested in (Fig. 3E). BTFPTU enhanced UPR and impaired cell cycle, especially G1 to S phase in GSEA analysis (Fig. S3A-F). The RNA-seq data fully demonstrated that BTFPTU induced transcriptional reprogramming in HOS cells, which may lead to cell death through multiple pathways, providing new directions for subsequent experiments.

### BTFPTU causes osteosarcoma cell death through multiple pathways in vitro

**Fig. 4:**
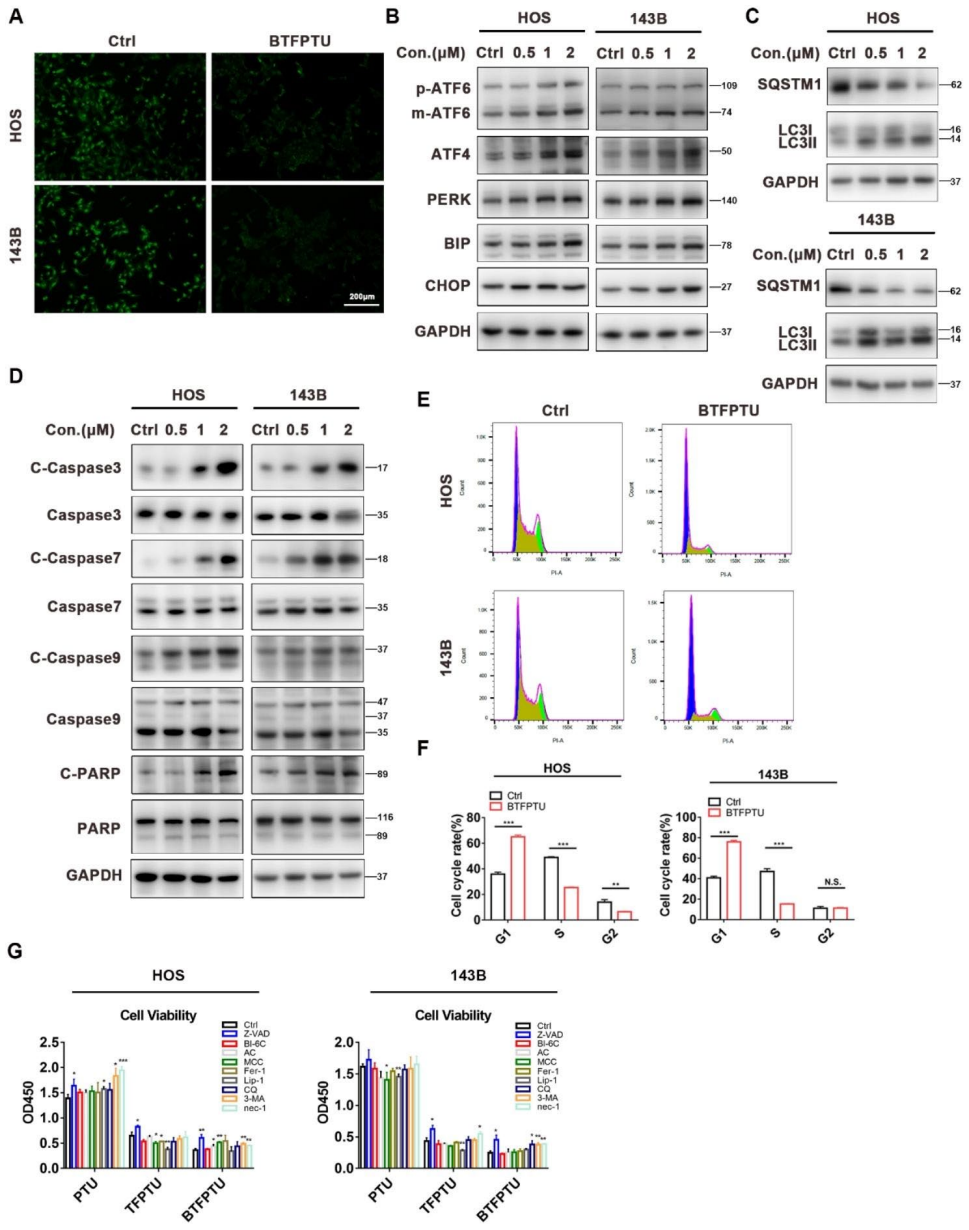
BTFPTU causes osteosarcoma cells death through multiple pathways in vitro. (**A**) Immunofluorescence of intracellular chloride ions in HOS cells treated with or without BTFPTU (2 µM) treatment for 6 h using MQAE staining. Scale bar, 200 μm. (**B**) The expression of ER stress proteins was measured by Western blot analysis in HOS and 143B cells treated with varying concentrations of BTFPTU for 24 h. (**C**) The expression of autophagy-related proteins was measured by Western blot analysis in HOS and 143B cells treated with varying concentrations of BTFPTU for 24 h. (**D**) The expression of apoptosis proteins was measured by Western blot analysis in HOS and 143B cells treated with varying concentrations of BTFPTU for 24 h. (**E**) Flow cytometry to detect cell cycle of HOS and 143B cells with or without BTFPTU (2 µM) treatment for 12 h by PI staining. (**F**) Quantification of different cell cycle phases of HOS and 143B cells. (**G**) Cell viability of HOS and 143B cells with Z-VAD (10 µM), BI-6 C (5 µM), AC (5 µM), MCC (10 µM), Fer-1 (5 µM), Lip-1 (2.5 µM), CQ (2.5 µM), 3-MA (2.5 mM) or nec-1 (5 µM) treatment under PTU, TFPTU or BTFPTU treatment for 24 h using Cell Counting Kit-8 assay. The data represent the mean ± SD of three independent experiments. *p < 0.05, **p < 0.01, ***p < 0.001 for a comparison with the control group or as indicated

Studies have shown that disruption of ion homeostasis affects diverse cellular functions, such as gene and protein expression and activities, post-translational modifications of proteins, cellular volume, cell cycle, cell proliferation and differentiation, membrane potential, reactive oxygen species levels, and intracellular/extracellular pH, leading to cell death [36, 37]. To investigate the mechanism by which BTFPTU induces cell death, we first examined whether it altered the ion homeostasis of osteosarcoma cells. Previous studies have shown that small-molecule ion transporters can transport ions driven by concentration gradients [38, 39], and Fig. 1F showed that BTFPTU had high transporting efficiency for chloride ions. Therefore, we used MQAE fluorescence staining to detect the intracellular chloride ion concentration. After 3 h of BTFPTU treatment, the influx of chloride ions caused a significant increase in the intracellular chloride ion concentration (MQAE fluorescence intensity is inversely proportional to the intracellular chloride ion concentration) (Fig. 4A). This suggested that BTFPTU could cause cell death by altering the ion homeostasis of cells.

By analyzing the RNA-seq data, we found that BTFPTU treatment activated multiple pathways leading to cell growth arrest or death in osteosarcoma cells. The relevant pathways involved in the RNA-seq data were further validated. Firstly, we verified the ER stress and autophagy pathways. The expression of ER stress marker proteins such as m-ATF6, ATF4, PERK, BIP, and CHOP were significantly upregulated, while the expression of autophagy-related protein SQSTM1 was downregulated and LC3B was upregulated (Fig. 4B, C and Fig. S4A, B). Similarly, after BTFPTU treatment, the expression levels of active forms of apoptotic proteins such as C-Caspase3, C-Caspase7, C-Caspase9, and C-PARP increased gradually with increasing BTFPTU concentration, while the expression levels of Caspase3, Caspase7, Caspase9, and PARP remained unchanged (Fig. 4D and Fig. S4C). Flow cytometry analysis of cell cycle proved that BTFPTU arrested osteosarcoma cells in the G1 phase and prevented them from entering the S phase (Fig. 4E, F). All of the above results are consistent with the RNA-seq data, indicating that BTFPTU inhibits cell proliferation or induces cell death by activating ER stress and autophagy pathways, disrupting the normal cell cycle, and increasing the expression of active forms of apoptotic proteins. Subsequently, the inhibitors of apoptosis (Z-VAD-FMK, BI-6C9), pyroptosis (Ac-YVAD-cmk, MCC950), ferroptosis (Ferrostatin-1, Liproxstatin-1), autophagy (Chloroquine, 3-Methyladenine), and necroptosis (Necrostatin-1) were applied to investigate which form of cell death inhibition can rescue osteosarcoma cells treated with BTFPTU. The CCK-8 results showed that only the pan caspase inhibitor, Z-VAD-FMK, had a partial rescue effect on cell viability (Fig. 4G). These results indicated that BTFPTU induced multiple modes of cell death in osteosarcoma cells by disrupting the ion homeostasis of cells, especially the chloride ion homeostasis.

### The self-assembled OTP-BTFPTU supramolecular liposomes inhibits the proliferation and migration of HOS cells in vitro

**Fig. 5:**
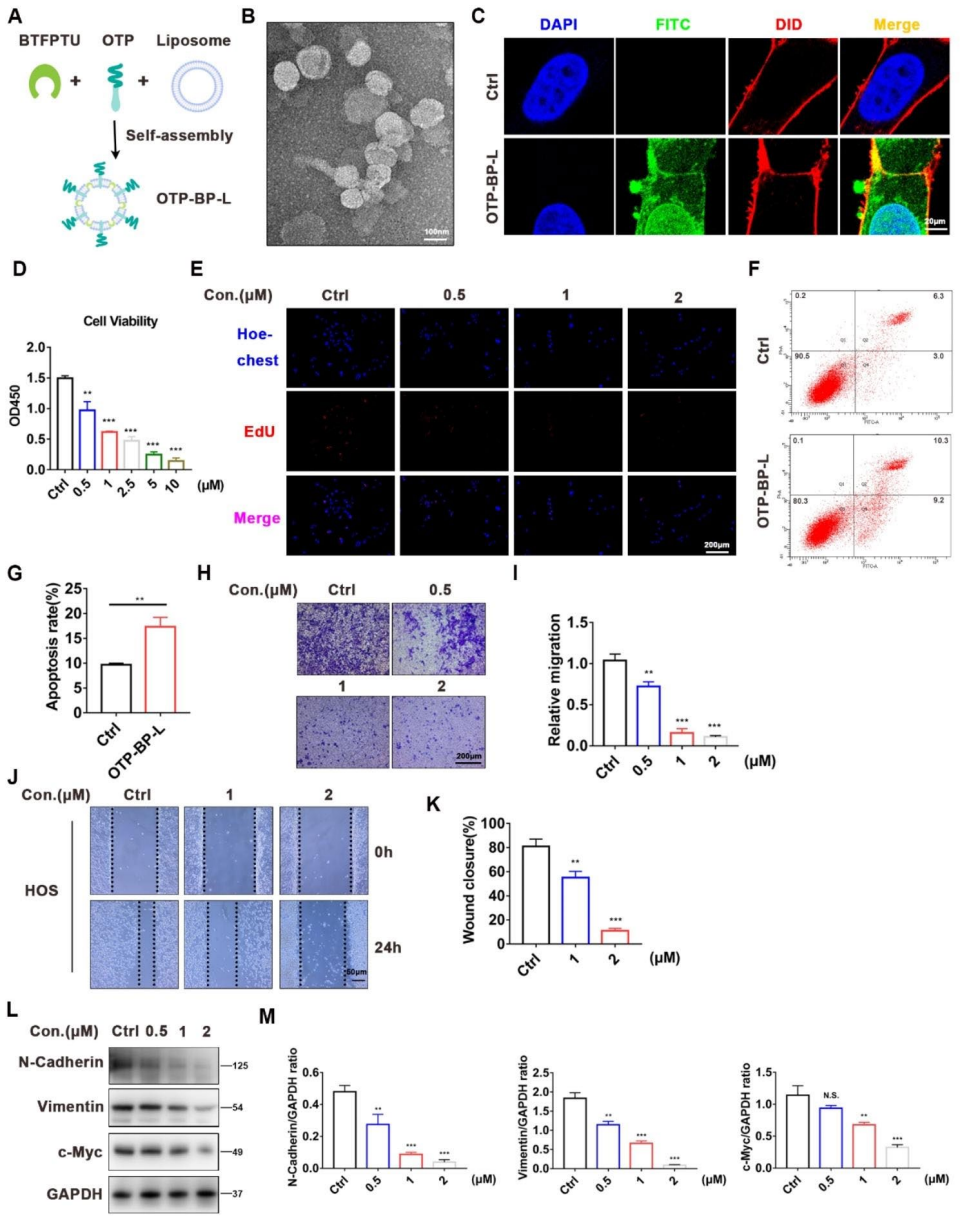
The self-assembled OTP-BTFPTU supramolecular liposomes inhibits the proliferation and migration of HOS cells in vitro. (**A**) Schematic illustration of osteosarcoma targeting peptide (OTP)-BTFPTU supramolecular liposomes prepared through self-assembly. (**B**) The TEM images of OTP-BTFPTU liposomes. Scale bar, 100 nm. (**C**) FITC (green), DID (red) and DAPI (blue) immunofluorescence of HOS cells with or without OTP-BTFPTU (2 µM) liposomes treatment for 3 h. Scale bar, 20 μm. (**D**) Cell viability of HOS cells with OTP-PTU, OTP-TFPTU or OTP-BTFPTU liposomes treatment for 24 h using Cell Counting Kit-8 assay. (**E**) HOS cells were treated with different concentrations of OTP-BTFPTU liposomes for 24 h and the cell proliferation was characterized by EdU assay. Scale bar, 200 μm. (**F**) Flow cytometry to evaluate apoptosis of HOS cells with or without OTP-BTFPTU liposomes (2 µM) treatment for 12 h by Annexin V-FITC/PI apoptosis detection. (**G**) Quantification of apoptosis rate of HOS cells. (**H**) Cell migration abilities of HOS cells treated with different concentrations of OTP-BTFPTU liposomes for 24 h were measured by Transwell migration assays. Scale bar, 200 μm. (**I**) Quantification of the migration abilities of HOS cells. (**J**) The wound healing assay was used to evaluate the migration abilities of HOS cells treated with different concentrations of OTP-BTFPTU liposomes for 24 h. Scale bar, 50 μm. (**K**) Quantification of the wound closure rate. (**L**) The protein expression of N-Cadherin, Vimentin and c-Myc was measured by Western blot analysis in HOS cells treated with varying concentrations of OTP-BTFPTU liposomes for 24 h. (**M**) Quantification and normalization of the gray levels of N-Cadherin, Vimentin and c-Myc proteins to that of GAPDH in HOS cells using Image J. The data represent the mean ± SD of three independent experiments. *p < 0.05, **p < 0.01, ***p < 0.001 for a comparison with the control group or as indicated

As BTFPTU is an ion transporter that can insert into the cell membranes and disrupt ion homeostasis, we designed a self-assembling complex to achieve targeted treatment of osteosarcoma lesions, which can reduce toxicity to other organs. The osteosarcoma targeting peptide (OTP, with the sequence of TPPRVPLLTFGS) was identified by phage display techniques, which can recognize and target osteosarcoma [31].We further modified the peptide by adding FITC and EDA-Tetradecanoic acid which allowed for better binding of the peptide to the lipid membrane. Then we mixed the modified OTP, BTFPTU, and liposomes (assembly scheme: 2 mg peptide + 646 µg BTFPTU + 10 mg liposomes per ml of solution) for self-assembly, as shown in Fig. 5A.

Transmission electron microscopy (TEM) revealed that the self-assembled osteosarcoma targeting peptide-BTFPTU supramolecular liposomes (OTP-BP-L) possessed a well-defined vesicular structure (Fig. 5B). Immunofluorescence results showed that OTP-BP-L had co-localization with cell membrane, indicating that OTP-BP-L can insert into the cell membrane and function as ion transporters (Fig. 5C). CCK-8 and EdU assays (Fig. 5D, E) shown that OTP-BP-L inhibited the proliferation of HOS cells, and flow cytometry proved that OTP-BP-L increased apoptosis in HOS cells (Fig. 5F, G). Similarly, after treatment with OTP-BP-L, the migration ability and expression levels of EMT marker proteins in HOS cells were significantly inhibited (Fig. 5H-M). To further confirm the toxic effects of OTP-BP-L on osteosarcoma cells, we measured the expression levels of apoptosis, ER stress, and autophagy-related proteins. Western blotting demonstrated that OTP-BP-L also could activate apoptosis, ER stress, and autophagy-related pathways, leading to cell death (Fig. S5A-F). These data confirmed that OTP-BTFPTU supramolecular liposomes inhibited the proliferation and migration of HOS cells in vitro.

### OTP-BTFPTU liposomes suppresses osteosarcoma tumorigenesis and metastasis in vivo

**Fig. 6:**
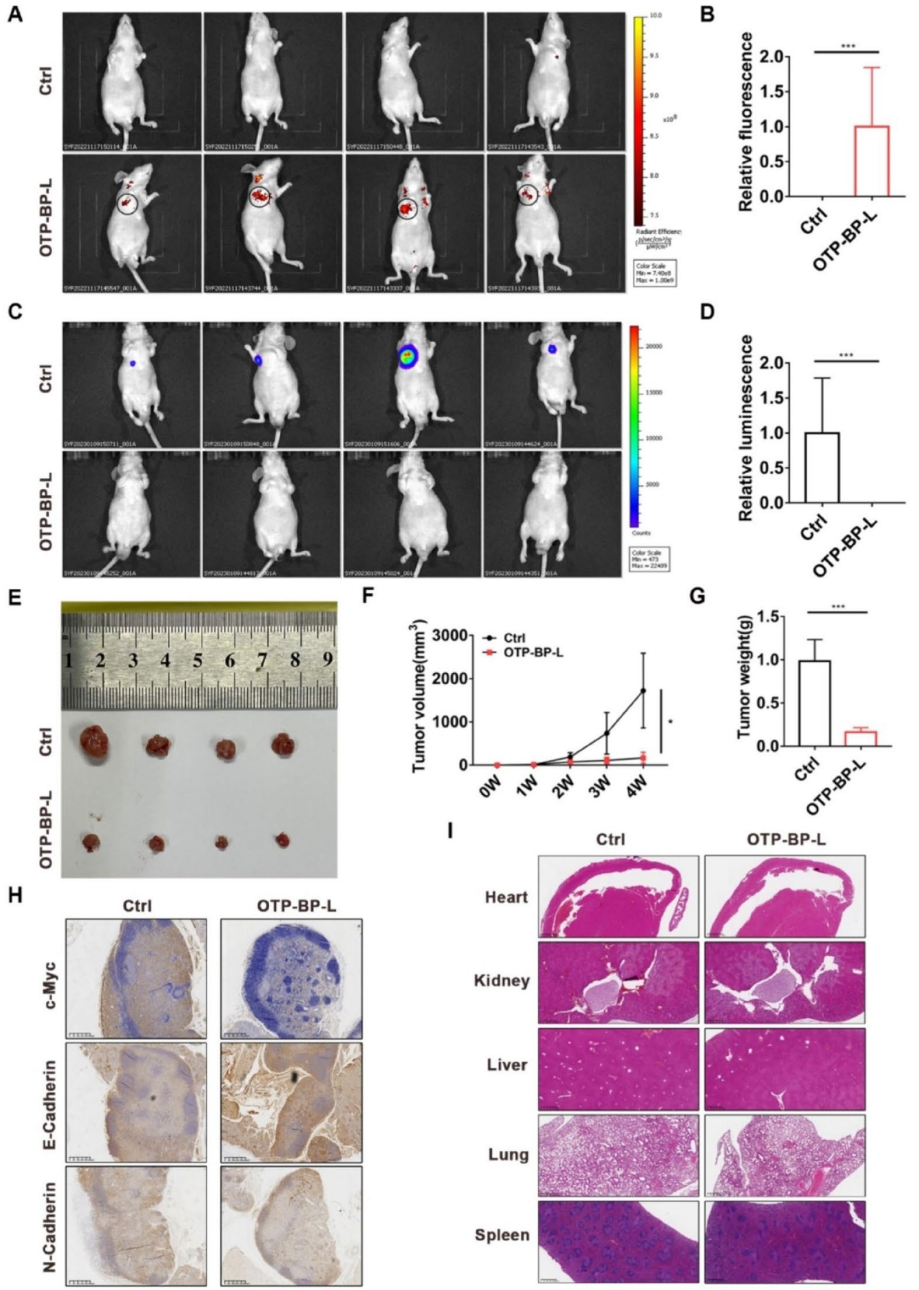
OTP-BTFPTU liposomes suppresses osteosarcoma tumorigenesis and metastasis in vivo. (**A**) Living fluorescence imaging of subcutaneous xenograft tumor model mice, which were injected with 0.1 mL of saline or OTP-BTFPTU liposomes (5 × 10 − 5 M) through tail vein. The imaging was performed 24 h after injection (n = 4). (**B**) Quantification of the relative fluorescence of the nude mice which were injected with saline or OTP-BTFPTU liposomes. (**C**) Living luminescence imaging of subcutaneous xenograft tumor model mice with saline or OTP-BTFPTU liposomes treatment (n = 4). (**D**) Quantification of the relative luminescence of subcutaneous xenograft tumor model mice with saline or OTP-BTFPTU liposomes treatment. (**E**) Photographs of HOS derived xenograft model with saline or OTP-BTFPTU liposomes treatment (n = 4). (**F**) Tumor volume of different groups was measured every 7 days after mice were injected with HOS cells. (**G**) The average tumor weight in each group when the mice were sacrificed. (**H**) The expression of c-Myc, E-cadherin and N-cadherin were determined by immunohistochemistry. Scale bars, 400 μm. (**I**) HE staining of heart, kidney, liver, lung and spleen which were obtained from different groups. Scale bars, 300 μm. The data represent the mean ± SD of three independent experiments. *p < 0.05, **p < 0.01, ***p < 0.001 for a comparison with the control group or as indicated

We have confirmed that the self-assembled OTP-BP-L can kill HOS cells in vitro. To further investigate its therapeutic effect on osteosarcoma in vivo, we established subcutaneous xenograft tumor and lung metastasis models with HOS cells stably transfected with luciferase reporter gene. To verify the tumor-homing ability of OTP-BP-L, we used in vivo fluorescence imaging to locate OTP-BP-L one day after intravenous injection. Strong fluorescence signals were detected in the subcutaneous tumor area, indicating that OTP-BP-L had good tumor-targeting ability and accumulated in tumor tissue (Fig. 6A, B). After 2 weeks of treatment with normal saline or OTP-BP-L, the luminescence signals in the subcutaneous tumor area of the OTP-BP-L treatment group were barely detectable, indicating that OTP-BP-L significantly inhibited subcutaneous tumor growth (Fig. 6C, D). Furthermore, OTP-BP-L treatment obviously decreased the tumor volume and weight compared to those in the control group (Fig. 6E-G). The expression of c-Myc and N-cadherin was reduced while the expression of E-cadherin was increased in tumor tissue by OTP-BP-L treatment (Fig. 6H). To evaluate the toxicity of OTP-BP-L to other organs, we isolated important organs such as the heart, kidney, liver, lung, and spleen, and performed HE staining. The results showed that OTP-BP-L treatment did not cause significant damage to these organs (Fig. 6I).

In the lung metastasis model, OTP-BP-L also reduced the luminescence signals and the size of lung metastatic foci (Fig. S6A-C). OTP-BP-L inhibited distant metastasis of osteosarcoma and reduced the expression of c-Myc and N-cadherin while increasing the expression of E-cadherin (Fig. S6D, E). These results indicated that OTP-BP-L had good targeting ability and therapeutic effect on osteosarcoma in situ lesions, and significantly inhibited distant metastasis of osteosarcoma in vivo.

### OTP-BTFPTU liposomes exhibit anti-drug resistance and can regulate the tumor immune microenvironment

**Fig. 7:**
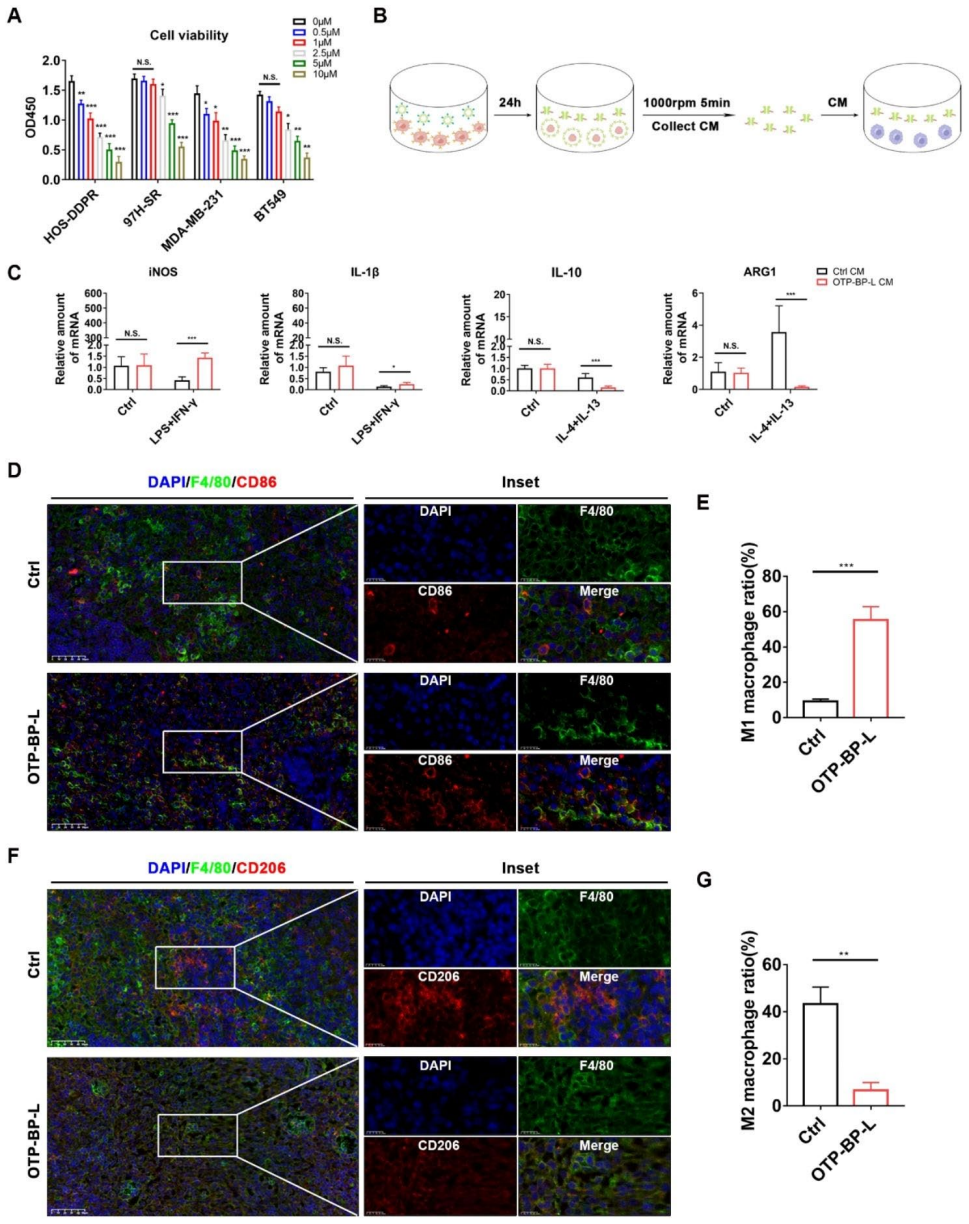
OTP-BTFPTU liposomes exhibit anti-drug resistance and can regulate the tumor immune microenvironment. (**A**) Cell viability of HOS-DDPR (cisplatin-resistant osteosarcoma cell line), 97 H-SR (sorafenib-resistant hepatoma cell line), MDA-MB-231 and BT549 (triple negative breast cancer cell lines) cells with OTP-BTFPTU liposomes treatment for 24 h using Cell Counting Kit-8 assay. (**B**) Schematic illustration of obtaining conditional medium (CM). (**C**) The mRNA level of iNOS, IL-1β, IL-10 and ARG1 in BMMs following M1 or M2 macrophage induction in the presence of 50% control or OTP-BTFPTU liposomes CM. (**D**) DAPI (blue), F4/80 (green) and CD86 (red) immunofluorescence of tumor sections from mice treated with or without OTP-BTFPTU liposomes. Scale bars, 50 μm. (**E**) Quantification of M1 macrophage (F4/80^+^＆CD86^+^) ratio. (**F**) DAPI (blue), F4/80 (green) and CD206 (red) immunofluorescence of tumor sections from mice treated with or without OTP-BTFPTU liposomes. Scale bars, 50 μm. (**G**) Quantification of M2 macrophage (F4/80^+^＆CD206^+^) ratio. The data represent the mean ± SD of three independent experiments. *p < 0.05, **p < 0.01, ***p < 0.001 for a comparison with the control group or as indicated

OTP-BP-L demonstrated good therapeutic effects on both in situ tumors and distant metastasis of osteosarcoma in vivo, so we wondered if OTP-BP-L was treating osteosarcoma only through its direct toxicity to osteosarcoma cells. Since osteosarcoma is prone to developing resistance to many drugs, which limits their therapeutic efficacy [40, 41], we investigated the anti-drug resistance of OTP-BP-L. Firstly, we treated strong drug resistance cell lines, such as cisplatin-resistant osteosarcoma cell line (HOS-DDPR), triple negative breast cancer cell lines (BT549 and MDA-MB-231) and sorafenib-resistant hepatoma cell line (MHCC-97 H-SR), with OTP-BP-L. The result showed that OTP-BP-L was still effective in killing drug resistance cell lines of osteosarcoma and other tumors (Fig. 7A).

In addition to the direct toxicity to tumor cells, the therapeutic effect of a drug on tumors in vivo also depends on its regulation of the tumor immune microenvironment. Tumor-associated macrophages (TAMs) are crucial for the development and progression of tumors, and it is generally believed that reprogramming of TAMs towards M1 phenotype can inhibit tumor growth [32, 33]. Based on this theory, we investigated the effect of OTP-BP-L-treated CM on the polarization of bone marrow-derived macrophages (BMMs) in vitro. HOS cells were treated with 0.5 µM OTP-BP-L for 24 h and the supernatant was collected after removing large cell fragments through centrifugation (Fig. 7B). The collected supernatant was then used as conditioned medium (CM) for BMMs culture. The qPCR results showed that OTP-BP-L-treated CM could increase the expression of CD86, iNOS, IL-1β, and IL-6, while inhibiting the expression of IL-10, ARG1, TGF-β, and CD206. These results indicated that the cell lysate products from OTP-BP-L treatment group promoted M1 polarization of macrophages and inhibited M2 polarization (Fig. 7C and Fig. S7A). To further validate the regulation of TAMs polarization by OTP-BP-L in vivo, we defined CD86^+^/F4/80^+^ cells as M1 macrophages and CD206^+^/F4/80^+^ cells as M2 macrophages according to Michael Klichinsky et al. [42], and performed immunofluorescence staining on tumor tissue sections. OTP-BP-L treatment significantly promoted M1 polarization and inhibited M2 polarization of TAMs (Fig. 7D-G). The results proved that OTP-BP-L could achieve its therapeutic effect by regulating the tumor immune microenvironment.

In summary, OTP-BP-L exhibited anti-drug resistance and ability to regulate the tumor immune microenvironment in vivo, which provided a new approach for the treatment of osteosarcoma.

## Discussion

Osteosarcoma is a highly malignant tumor that commonly occurs in children and adolescents, and is characterized by a high propensity for distant metastasis, drug resistance and high heterogeneity, with a lack of targeted treatment strategies. As a result, the treatment of osteosarcoma has been a major challenge in clinical treatment [5, 43]. In this study, we addressed several major issues in osteosarcoma treatment by using a synthetic transmembrane ion transporter (BTFPTU). By loading ion transporters and osteosarcoma-targeting peptides (OTP) into liposomes, the self-assembled supramolecular drug can target and eliminate osteosarcoma lesions, resist drug resistance, and regulate the immune microenvironment in vivo.

We have synthesized three ion transporters based on O-bridged scaffold with different functional groups. As shown in our previous study, the ion transport activity increases when adding electron-withdrawing groups such as -CF_3_ to the transporters [34]. For example, when modified with two -CF_3_ groups instead of one, the EC50 value (effective concentration needed to achieve half of the chloride influx after addition of transporter) increased from 0.041 mol% for TFPTU to 0.0045 mol% for BTFPTU. We screened three types of ion transporters, PTU, TFPTU, and BTFPTU, and found that BTFPTU had the best therapeutic efficacy against osteosarcoma in vitro.

Over the past 40 years, researchers have been exploring the mechanisms of chemoresistance in osteosarcoma, including drug inactivation, changes in drug targets, drug efflux, epigenetic changes, EMT, and inhibition of cell death. Although understanding of chemoresistance in osteosarcoma has deepened, effective methods to address this issue are still lacking. The current consensus is that tumor cells are more likely to develop resistance to the drug which causes a single mode of cell death [44–46]. BTFPTU is a transmembrane ion transporter that can insert into the cell membrane without the need for recognition sites or intracellular metabolic processes, and disrupt intracellular ion homeostasis, especially chloride ion homeostasis. Imbalance of intracellular chloride ion homeostasis affects diverse cellular functions and ultimately leads to cell death [36, 37]. Due to its direct binding to the cell membrane, BTFPTU is difficult to be expelled from cells unless the cells die. These characteristics determine that osteosarcoma cells are unlikely to develop resistance to this type of ion transporters through drug inactivation, changes in drug targets, or drug efflux. Although previous studies have also investigated the effects of ion transporters on cells in vitro, these studies have only explored the induction of cell death through apoptosis. Our research has, for the first time, revealed that this class of ion transporters can induce cell death through various mechanisms, including activation of ER stress, autophagy, and cell cycle arrest. Furthermore, BTFPTU-induced multi-mode cell death was less likely to induce drug resistance in osteosarcoma cells compared to single- mode cell death.

The high heterogeneity of osteosarcoma has led to the lack of targeted drugs for clinical treatment. Despite the heterogeneity of tissues or organs, phage display techniques can still identify tissue or organ specific binding peptides [47]. Furthermore, compared to targeted drugs such as antibodies, targeted peptides selected by phage display have more precise binding sites [48]. Lin et al. used phage display techniques to screen for a peptide, which only had recognition function for osteosarcoma and didn’t have killing effect on tumor cells [31]. Since the identification of this peptide was based on HOS cells, we used HOS cells for subsequent studies as well. We utilized this OTP and modified it with FITC and EDA-Tetradecanoic acid to improve its binding to the lipid membrane. BTFPTU, OTP and liposomes self-assembled into a supramolecular drug (OTP-BP-L) which used for the treatment of osteosarcoma. OTP-BP-L had good targeting ability and therapeutic efficacy against osteosarcoma in situ lesions in vivo, and could significantly inhibit distant metastasis of osteosarcoma. The toxicity to other organs under systemic application of OTP-BP-L was also considered, and experiments showed that OTP-BP-L didn’t cause significant damage to important organs during treatment. To further apply OTP-BP-L in vivo, the retention time in tumor tissue and metabolic processes of OTP-BP-L will be the focus of our future research.

Further studies on OTP-BP-L showed that it had good killing effects on drug resistant tumor cell lines, indicating that OTP-BP-L also has good anti-resistance properties. In vivo application is different from cell culture in vitro because the lysate products after cell death have a crucial impact on the tumor tissue and its microenvironment. Unlike tumor cells, stromal cell types within the tumor microenvironment (TME) are genetically stable and thus represent an attractive therapeutic target with reduced risk of resistance and tumor recurrence [49]. Tumor-associated macrophages (TAMs) are an essential component of the tumor microenvironment and have a role in the orchestration of various processes, including resistance to chemotherapeutic agents and checkpoint blockade immunotherapy [32]. The in vitro and in vivo experiments indicated that OTP-BP-L treatment remodeled the tumor immune microenvironment, promoting the polarization of TAMs towards the M1 phenotype that inhibited tumor growth.

In summary, the self-assembly osteosarcoma targeting peptide-BTFPTU supramolecular liposomes had good tumor targeting capability, exhibited anti-drug resistance and regulated the tumor immune microenvironment. This work not only demonstrated the biomedical value of small-molecule anion transporters in vivo, but also provided an innovative approach for the treatment of osteosarcoma.

## Conclusion

In summary, we have demonstrated for the first time that small-molecule anion transporters are capable of killing osteosarcoma cells through multiple pathways, rather than solely relying on the previously reported caspase-dependent apoptotic pathway. Through a co-assembly process, we have successfully prepared supramolecular drugs by loading anion transporters into osteosarcoma targeting peptide functionalized liposomes. The assemblies, OTP-BP-L, show excellent targeting and therapeutic effect towards osteosarcoma tumors. Furthermore, the supramolecular drug shows a strong ability to regulate the tumor immune microenvironment in subcutaneous xenograft tumor and lung metastasis models. This work not only demonstrated the biomedical value of small-molecule anion transporters in vivo, but also provided an innovative approach for the treatment of osteosarcoma.

## Methods

### Dynamic light scattering

The size of the LUVs was characterized by dynamic light scattering (DLS) using a NanoBrook Omni (Brookhaven Instruments Corporation). A laser wavelength of 659 nm and a scattering angle of 90° were used.

### Cationic selectivity

Egg yolk phosphatidylcholine was dissolved in CH_3_Cl, (10 mg/mL, Shanghai Macklin Biochemical, China). The lipid solution was evaporated to form a thin film by purging N_2_ slowly. After drying the resulting film under high vacuum overnight at room temperature, the film was hydrated with 4-(2- hydroxyethyl)-1-piperazine-ethane sulfonic acid (HEPES) buffer solution (1 mL, 25 mM HEPES, 100 mM MCl, M = Li^+^, Na^+^, K^+^, Cs^+^, pH = 7.0) containing a pH sensitive dye 8-hydrox-ypyrene-1,3,6-trisulfonic acid (HPTS, 0.2 mM) at 40℃ for 2 h to give a milky suspension. The mixture was then subjected to 10 freeze-thaw cycles (freezing in liquid N_2_ for 1 min and thawing at water bath for 2 min). The vesicle suspension was extruded through polycarbonate membrane (0.2 μm) to produce a homogeneous suspension of LUVs of about 200 nm in diameter with HPTS encapsulated inside. The extravesicular HPTS dye was removed by using size exclusion chromatography (stationary phase: Sephadex G-50, Shanghai Macklin Biochemical, China, mobile phase: HEPES buffer with 100 mM NaCl) and diluted with the mobile phase to yield 3 mL lipid stock solution.

100 µL of HPTS-containing LUVs and 10 µL PTU/TFPTU/BTFPTU in a certain concentration were added to 900 µL of HEPES buffer (25 mM HEPES, 100 mM MCl, M = Li^+^, Na^+^, K^+^, Cs^+^, pH = 7.0) in a clean fluorescence cuvette. Then mix the above solution evenly. This cuvette was placed on the fluorescence instrument (at t = 0 s). Fluorescence emission intensity of HPTS was monitored. 10 µL of 5 M MOH (M = Li^+^, Na^+^, K^+^, Cs^+^) was added at t = 20 s to generate pH gradient across lipid bilayer and recorded simultaneously for 300 s using fluorescence spectrophotometer (Hitachi, F-4700, Japan). Finally at t = 320 s, 10µL of 10% Triton X-100 was added to destroy all vesicles which resulted in destruction of pH gradient to achieve the maximum change in fluorescence emission intensity of HPTS dye.

### Anionic selectivity

Egg yolk phosphatidylcholine was added in CH_3_Cl. (10 mg/mL, Shanghai Macklin Biochemical, China). The lipid was evaporated by purging N_2_ slowly. After drying the resulting film under high vacuum overnight at room temperature, the film was hydrated with HEPES buffer solution (1 mL, 25 mM HEPES, 50 mM Na_2_SO_4_/100 mM NaX, X = HCO_3_^−^, NO_3_^−^, Cl^−^, Br^−^, pH = 7.0, except NaHCO_3_ buffer pH = 7.3) containing 0.2 mM HPTS at 40℃ for 2 h to give a milky suspension. The mixture was then subjected to 10 freeze-thaw cycles (freezing in liquid N_2_ for 1 min and thawing at water bath for 2 min). The vesicle suspension was extruded through polycarbonate membrane (0.2 μm) to produce a homogeneous suspension of large unilamellar vesicles (LUVs) of about 200 nm in diameter with HPTS encapsulated inside. The extravesicular HPTS dye was removed by using size exclusion chromatography (stationary phase: Sephadex G-50, Shanghai Macklin Biochemical, China, mobile phase: HEPES buffer with 100 mM NaCl) and diluted with the mobile phase to yield 3 mL lipid stock solution.

100 µL of HPTS-containing LUVs and 10 µL PTU/TFPTU/BTFPTU in a certain concentration were added to 900 µL of HEPES buffer (25 mM HEPES, 50 mM Na_2_SO_4_/100 mM NaX, X = HCO_3_^−^, NO_3_^−^, Cl^−^, Br^−^, pH = 7.0, except NaHCO_3_ buffer pH = 7.3) in a clean fluorescence cuvette. Then mix the above solution evenly. This cuvette was placed on the fluorescence instrument (at t = 0s). Fluorescence emission intensity of HPTS was monitored over time. 10 µL of 5 M NaOH was added at t = 20 s to generate pH gradient across lipid bilayer and recorded simultaneously for 300 s using fluorescence spectrophotometer (Hitachi, F-4700, Japan). Finally at t = 320 s, 10 µL of 10% Triton X-100 was added to destroy all vesicles which resulted in destruction of pH gradient to achieve the maximum change in fluorescence emission intensity of HPTS dye.

### Sample pretreatment for TEM

The thin pure carbon film coated grids (400 mesh, Zhongjingkeyi Technology Co., Ltd) were firstly hydrophilic treated applying sample. Grids were first placed on a clean slide (carbon-coated side is facing up), then moved into ion sputter instrument and discharge treated under vacuum condition for 8 s (~ 5 Pa, 10 ~ 15 mA).

Three drops of UranyLess EM stain (Haide Chuangye (Beijing) Biotechnology Co., Ltd.) were applied on the surface of parafilm in advance. The processed grid was clamped with self-locking tweezer (carbon-coated side is facing up) and 3 µL sample solution was placed on it, adsorbed for 30 s, then liquid was blotted up with filter paper and the grid was immediately inserted into a drop of uranyless EM stain (carbon-coated side is facing down) and gently shaken for 10 s, blotted up with filter paper and inserted into the second drop of uranyless EM stain, stained for 10 s, blotted up with filter paper. Finally the grid was inserted into the third drop of uranyless EM for 1 min prior to blotting up with filter paper and further dried in dryer. The stained sample was characterized with JEM 1400 Plus (JEOL) with an acceleration voltage of 120 kv.

### Cell culture

Two human OS cell lines, including HOS (ATCC: CRL-1543) and 143B (ATCC: CRL-8303) were purchased from the (ATCC, Manassas, VA, USA). Triple negative breast cancer cell lines (BT549 and MDA-MB-231) were provided by Prof. Jun Zhang (Clinical Laboratory, Sir Run Run Shaw Hospital, Zhejiang, China) and Sorafenib-resistant hepatoma cell line (MHCC-97 H-SR) was provided by Prof. Xian Wang (Department of oncology, Sir Run Run Shaw Hospital, Zhejiang, China). HOS, 143B, MDA-MB-231 and MHCC-97 H were cultured in Dulbecco’s modified Eagle’s medium (DMEM) and BT549 was cultured in 1640 medium containing 10% fetal bovine serum (FBS, Gibco, Gaithersburg, MD, USA) with 5% CO_2_ at 37℃.

### Colony-formation assay

HOS and 143B cells were trypsinized and seeded at a density of 3 × 10^3^ cells/well into a 12-well plate. After 5 days of culture with treatment, the cells were washed with phosphate-buffered saline (PBS) and fixed with 4% paraformaldehyde for 30 min. The fixed colonies were stained with 1% crystal violet solution for 10 min at room temperature. The colonies were imaged and counted under a microscope after washed by PBS.

### Wound-healing assay

We prepared the Culture-insert (80,209, ibidi, Germany) and seeded HOS or 143B cells in 24-well plates. Culture-insert was removed after 24 h cell culture. Subsequently, HOS or 143B cells were cultured with treatment for 24 h and the cells were washed twice with PBS. The images of cell-free gap from the same position were captured by microscopy at 0 and 24 h. The ratio of the area of wound-healing was quantified by Image J software (NIH, Bethesda, MD, USA).

### Transwell migration assay

We used 24-well transwell chambers (140,644, Thermo Scientific, USA) to evaluate migration capability of HOS and 143B cells. Cells were resuspended with FBS-free DMEM and seeded into upper chambers (5 × 10^4^ cells/well). 500 µL DMEM containing 20% FBS was added to the lower chambers. Following 24 h of culture with different treatment, cells in the upper chamber were removed and the lower side of the chamber was gently washed twice with PBS and fixed with 4% paraformaldehyde for 30 min at room temperature. Cells were stained with 1% crystal violet solution for 10 min and washed by PBS for five times. Cells which detained in the upper side were wiped off and images were captured by microscopy.

### EdU assay

HOS or 143B cells were seeded in 24-well plates (5 × 10^4^ cells/well) and cultured with different treatment. After 24 h, cells were staining with EdU and Hoechst dye according to the instructions (C10310-1, Cell-Light EdU Apollo567 In Vitro Kit, RIBOBIO, China).

### Apoptosis and cell cycle analysis

HOS or 143B cells were seeded into 12-well plates (2 × 10^5^ cells/well) and cultured with different treatment for 24 h after they were adherent. For cell apoptosis assay or cell cycle analysis, cells were trypsinized, centrifuged, washed with ice-cold PBS and stained with Annexin V-FITC/PI (40,302, YEASEN, China) or PI (40,301, YEASEN, China) for 15–30 min according to the instructions. Finally, the different apoptosis period and cell cycle of cells were analyzed by a flow cytometer (BD FACSCANTO II, BD Biosciences, USA).

### Subcutaneous xenograft tumor and lung metastasis models

Nude mice (male, 4-week-old) was provided by the Animal Center of the Sir Run Run Shaw Hospital (Zhejiang, China). Subcutaneous xenograft tumor or lung metastasis model was established with a total of 1 × 10^7^ HOS cells, which stably transfected with luciferase reporter gene, in 0.1 mL of PBS injected into the right flank of the mice or tail vein. After 2 weeks, the tumor-bearing mice of subcutaneous xenograft tumor or lung metastasis model were divided into two groups randomly, which were injected with 0.1 mL of saline or OTP-BTFPTU liposomes (1 × 10^− 5^ M) twice a week via tail vein. After 2 weeks treatment, the tumor-bearing mice were performed with live imaging and sacrificed to obtain the tumor. Tumor volume was calculated by the equation v = ab^2^/2.

### In vivo fluorescence imaging of OTP-BTFPTU liposomes

After 2 weeks of tumor formation, 0.1 mL of saline or OTP-BTFPTU liposomes (1 × 10^− 5^ M) was injected to tumor-bearing mouse through tail vein. All mice were anesthetized with a 5% isoflurane/oxygen mixture and scanned under in vivo Imaging System (Ex/Em:493/517 nm) (IVIS Lumina LT, PerkinElmer, USA) 24 h after intravenous injection.

### Histology assay

The osteosarcoma, lung, heart, kidney, liver, spleen samples were fixed in 4% paraformaldehyde at room temperature for 2 days. The slices of about 4 μm were used for H&E (hematoxylin and eosin) staining and immunohistochemical staining. We performed immunohistochemical staining for c-Myc (sc-40, Santa Cruz, 1:100), E-Cadherin (EM0502, HUABIO, 1:100) and N-Cadherin (ET1607-37, HUABIO, 1:100) according to the formerly protocol [50]. As for immunofluorescence staining, the primary antibody of F4/80 (sc-377,009, Santa Cruz, 1:100), CD86 (ET1606-50, HUABIO, 1:100) and CD206 (ab64694, Abcam, 1:100) were applied at 4 °C overnight. The secondary antibody of Goat anti-Rabbit IgG (H + L) Secondary Antibody (Alexa Fluor 555) (A31572, Invitrogen, 1:400) and Goat anti-Mouse IgG (H + L) Secondary Antibody (Alexa Fluor 488) (A11001, Invitrogen, 1:400) were applied at room temperature for 2 h.

### Western blot analysis

HOS and 143B cells were plated (5 × 10^5^ cells/well) in 6-well plates. Cells were cultured with different treatment for indicated time. RIPA (Solarbio, Beijing, China) mixed with 100mM phenylmethanesulfonyl fluoride (Beyotime, Zhengzhou, China), Protease Inhibitor Cocktail (Millipore, USA) and Phosphatase Inhibitor Cocktail (CWBIO, China) were used to extract the proteins. The extract was centrifuged for 10 min at 12,000 rpm under 4℃ and the obtained supernatants were heated for 10 min at 100℃. For Western blotting, we performed SDS–PAGE (10%) according to the previously described protocol [51]. The protein bands were visualized by Amersham Imager 600 (GE, USA). Image J was employed to analyze the images. Following antibodies were used for Western blotting: Apoptosis Antibody Sampler Kit (9915, Cell Signaling Technology, 1:1000), LC3 Rabbit Antibody (2775, Cell Signaling Technology, 1:1000), SQSTM1 Rabbit Antibody (39,749, Cell Signaling Technology, 1:1000), ATF4 Rabbit Antibody (ET1612-37, HUABIO, 1:1000), ATF6 Mouse Antibody (sc-166,659, Santa Cruz, 1:1000), PERK Rabbit Antibody (ab229912, Abcam, 1:1000), BiP Rabbit Antibody (3177, Cell Signaling Technology, 1:1000), CHOP Rabbit Antibody (ET1703-05, HUABIO, 1:1000), GAPDH Mouse Antibody (AC002, ABclonal, 1:1000).

### Cellular immunofluorescent staining

DiD Perchlorate (D8700, Solarbio, China) was used for cell membrane staining according to the instructions. In brief, cells were incubated with 5 µM dye solution at 37℃ for 20 min and washed with DMEM for 3 times. The images were taken by confocal microscopy (Olympus Corporation, Japan) (Ex/Em:644/663nm).

### Statistical analysis

All data were represented as mean ± S.D. SPSS 19.0 (SPSS, Chicago) served for statistical analyses. Statistical differences were analyzed by two-tailed Student’s t-test or one-way ANOVA followed by Tukey’s post hoc analyses where appropriate. P-value ≤ 0.05 was considered statistically significant as indicated in the figure legend.

### Electronic supplementary material

Below is the link to the electronic supplementary material.

**Supplementary Material 1:** Supplementary material of Figs. S1–S7

## Data Availability

All data needed to evaluate the conclusions in the paper are present in the paper and/or the Supplementary Materials. Additional data related to this paper are available from the authors upon reasonable request.

## List of abbreviations

ER: Endoplasmic reticulum
ROS: Reactive oxygen species
ALP: Alkaline phosphatase
TAMs: Tumor-associated macrophages
OTP: Osteosarcoma-targeting peptides
HPTS: 8-hydroxypyrene-1,3,6-trisulfonic acid
EYPC: Egg yolk phosphatidylcholine
EMT: Epithelial-mesenchymal transition
RNA-seq: RNA sequencing
UPR: Unfolded protein response
KEGG: Kyoto encyclopedia of genes and genomes
GO: Gene Ontology
OTP-BP-L: osteosarcoma targeting peptide-BTFPTU supramolecular liposomes
DLS: Dynamic light scattering
LUVs: Large unilamellar vesicles
PBS: Phosphate-buffered saline
