# Supplementary material for: In vivo therapy of osteosarcoma using anion transporters-based supramolecular drugs
Source: J Nanobiotechnology. 2024 Jan 13;22:29. doi: 10.1186/s12951-023-02270-x (PMC10787436; doi:10.1186/s12951-023-02270-x)
Supplement: Supplementary file 1 — Supplementary Material 1: Supplementary material of Figs. S1–S7 [file 12951_2023_2270_MOESM1_ESM.docx]

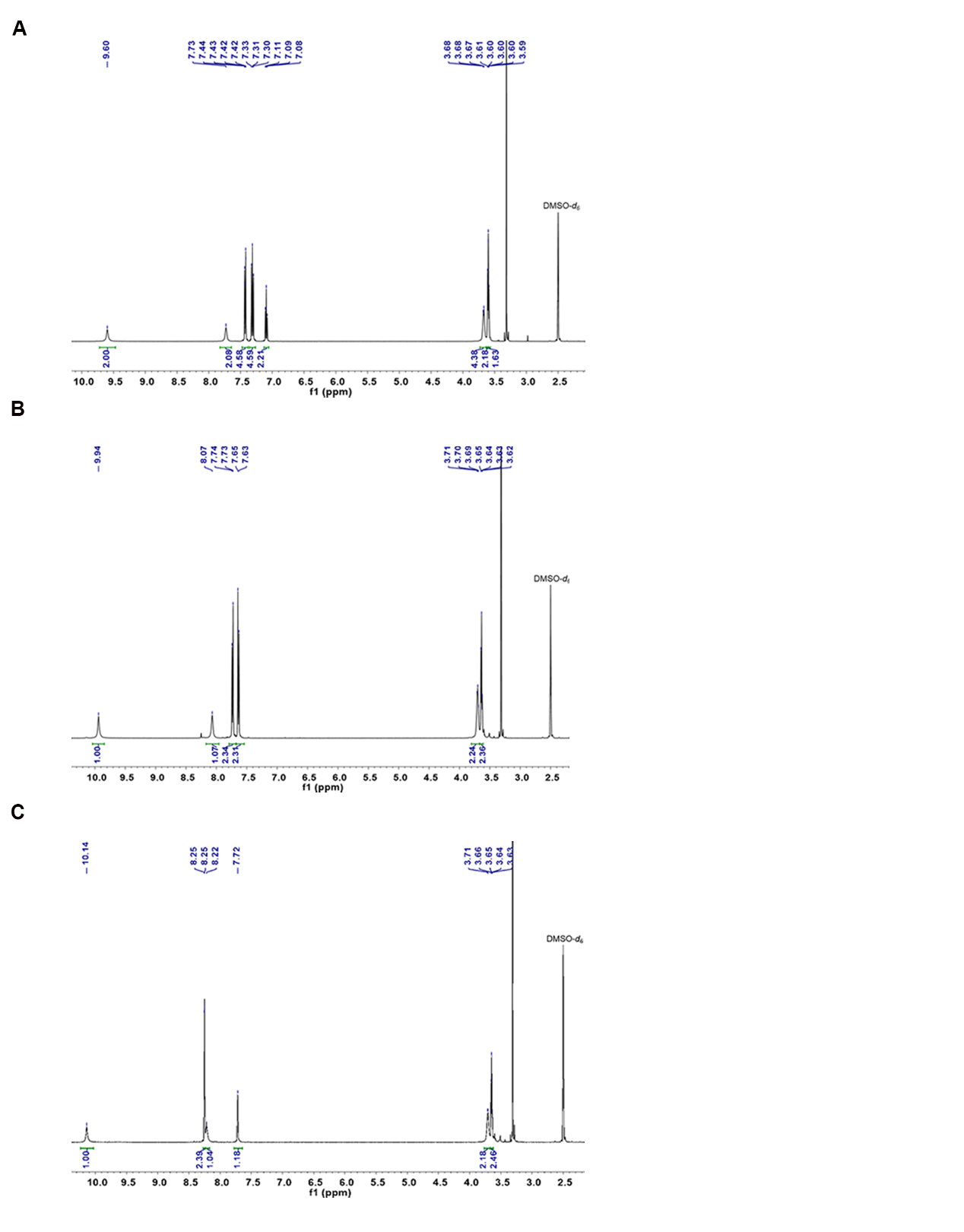


**Fig. S1 ^1^H NMR spectrum of ion transports. (A-C)** ^1^H NMR spectrum of PTU, TFPTU and BTFPTU in DMSO-*d6*.
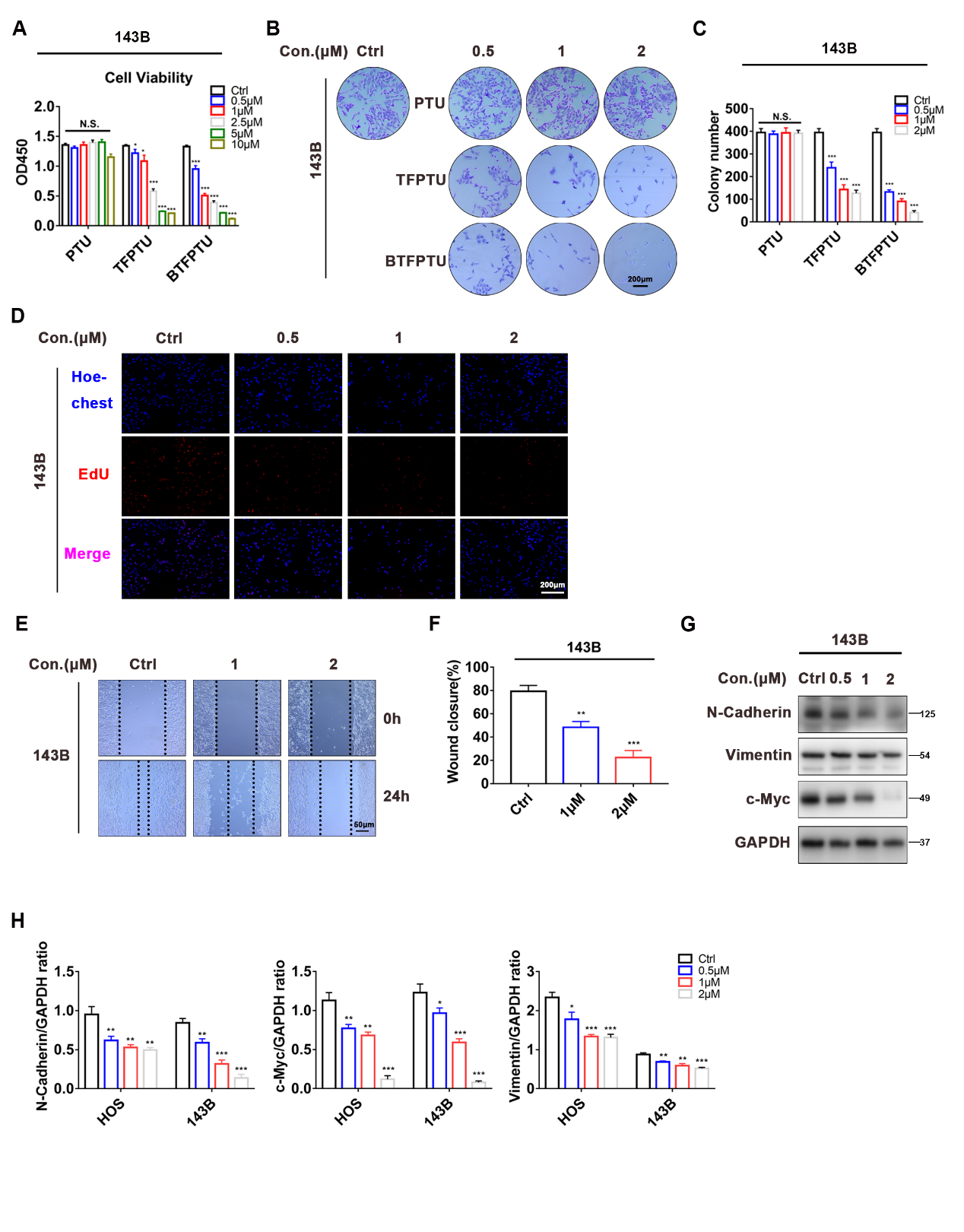


**Fig. S2 BTFPTU inhibits the proliferation and migration of 143B cells. (A)** Cell viability of 143B cells with PTU, TFPTU or BTFPTU treatment for 24 h using Cell Counting Kit-8 assay. **(B)** Effect of treatment with PTU, TFPTU or BTFPTU for 48 h on the colony-forming ability of 143B cells based on colony formation assays. Scale bar, 200 μm. **(C)** Quantification of the colony number. **(D)** 143B cells were treated with different concentrations of BTFPTU for 24 h and the cell proliferation was detected by EdU assay. Scale bar, 200 μm. **(E)** The wound healing assay was used to evaluate the migration abilities of 143B cells treated with different concentrations of BTFPTU for 24 h. Scale bar, 50 μm. **(F)** Quantification of the wound closure rate. **(G)** The protein expression of N-Cadherin, Vimentin and c-Myc was measured by Western blot analysis in 143B cells treated with varying concentrations of BTFPTU for 24 h. **(H)** Quantification and normalization of the gray levels of N-Cadherin, c-Myc and Vimentin proteins to that of GAPDH in HOS and 143B cells using Image J. The data represent the mean ± SD of three independent experiments. *p < 0.05, **p < 0.01, ***p < 0.001 for a comparison with the control group or as indicated.


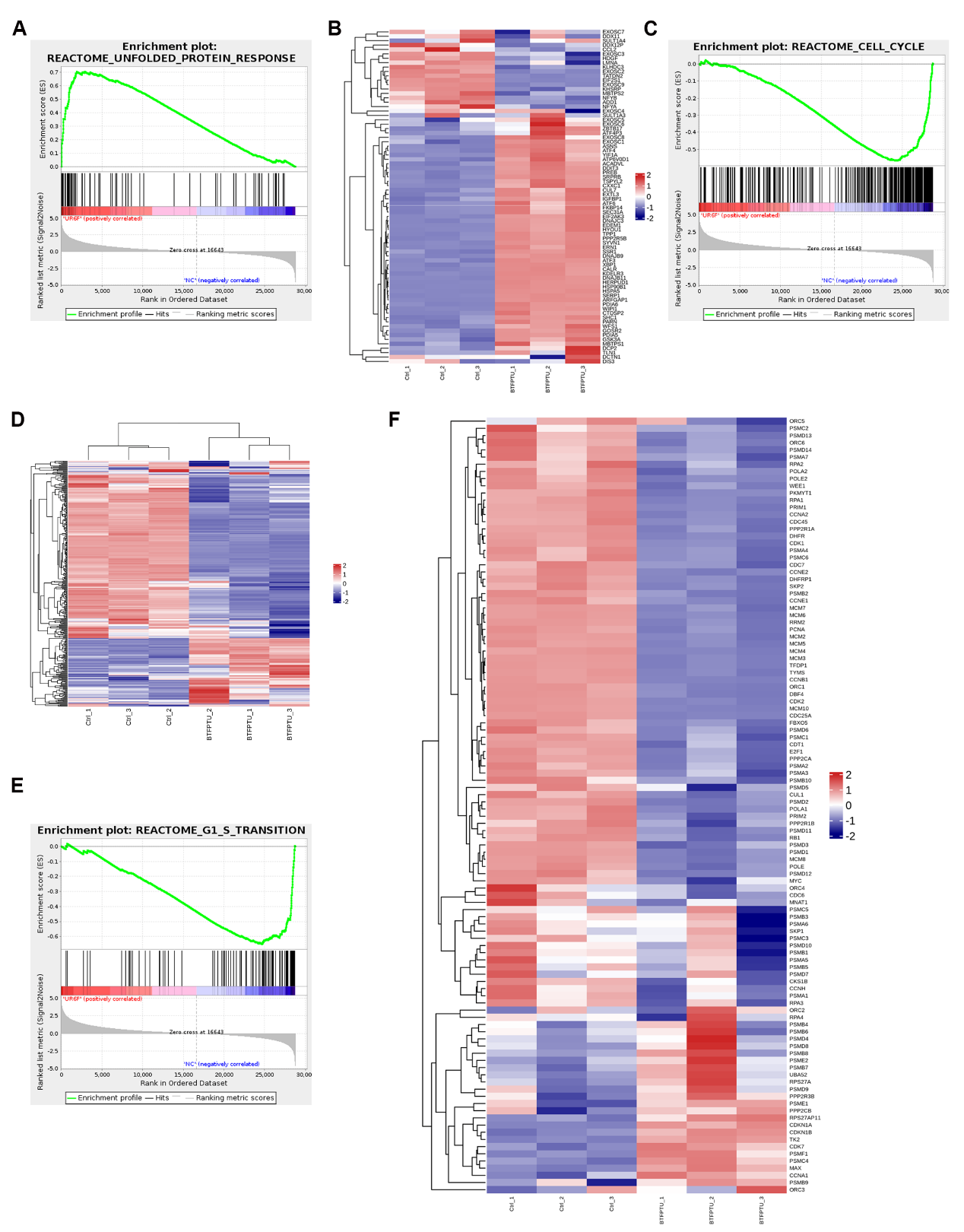


**Fig. S3 BTFPTU triggers transcriptional reprogramming of HOS cells. (A)** Enrichment plot of unfolded protein response. **(B)** GSEA of unfolded protein response. **(C)** Enrichment plot of cell cycle. **(D)** GSEA of cell cycle. **(E)** Enrichment plot of G1 to S transition. **(F)** GSEA of G1 to S transition.
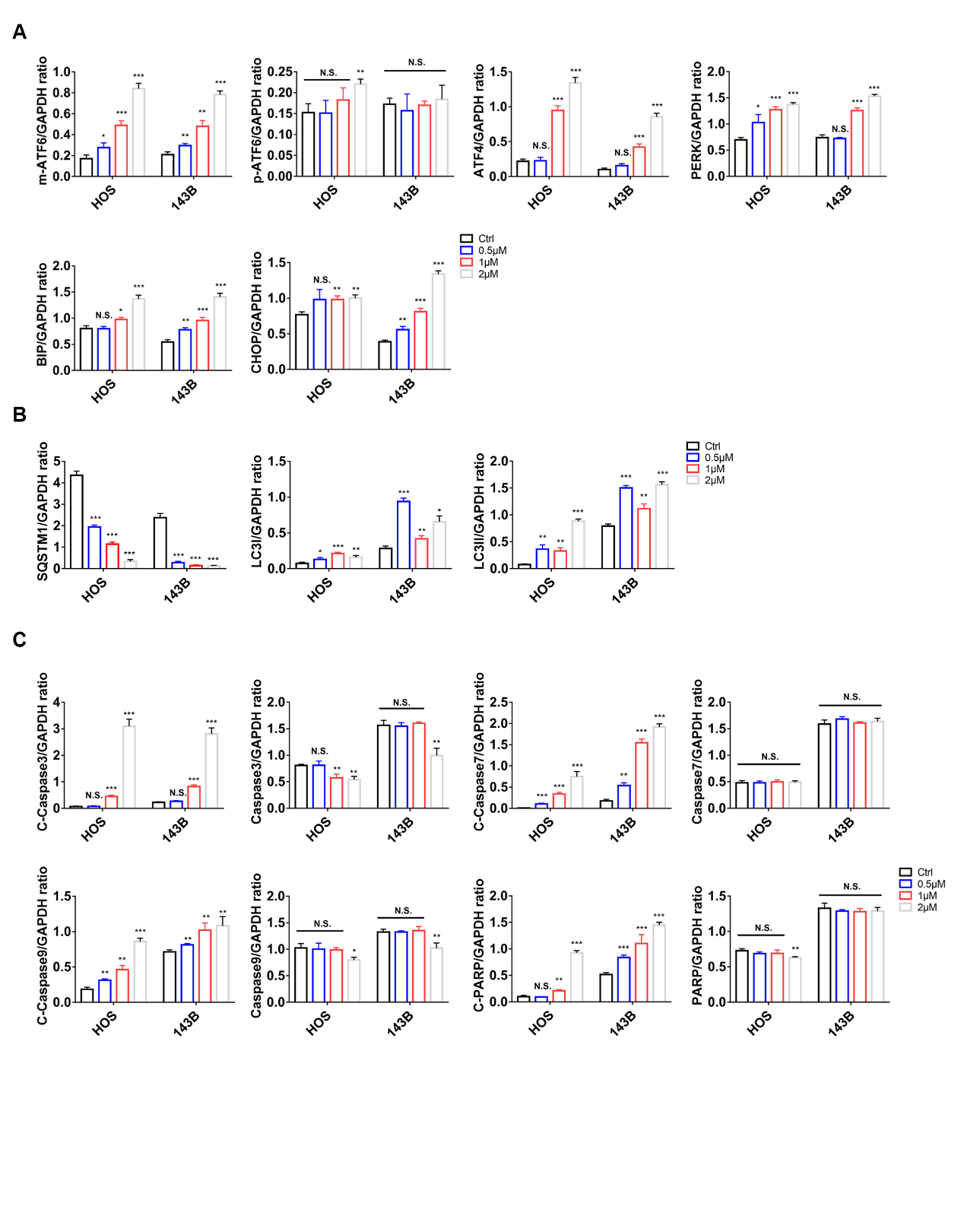


**Fig. S4 BTFPTU causes osteosarcoma cells death through multiple pathways in vitro. (A)** Quantification and normalization of the gray levels of p-ATF6, m-ATF6, ATF4, PERK, BIP and CHOP proteins to that of GAPDH in HOS and 143B cells using Image J. **(B)** Quantification and normalization of the gray levels of SQSTM1, LC3I and LC3II proteins to that of GAPDH in HOS and 143B cells using Image J. **(C)** Quantification and normalization of the gray levels of C-Caspase3, Caspase3, C-Caspase7, Caspase7, C-Caspase9, Caspase9, C-PARP and PARP proteins to that of GAPDH in HOS and 143B cells using Image J. The data represent the mean ± SD of three independent experiments. *p < 0.05, **p < 0.01, ***p < 0.001 for a comparison with the control group or as indicated.


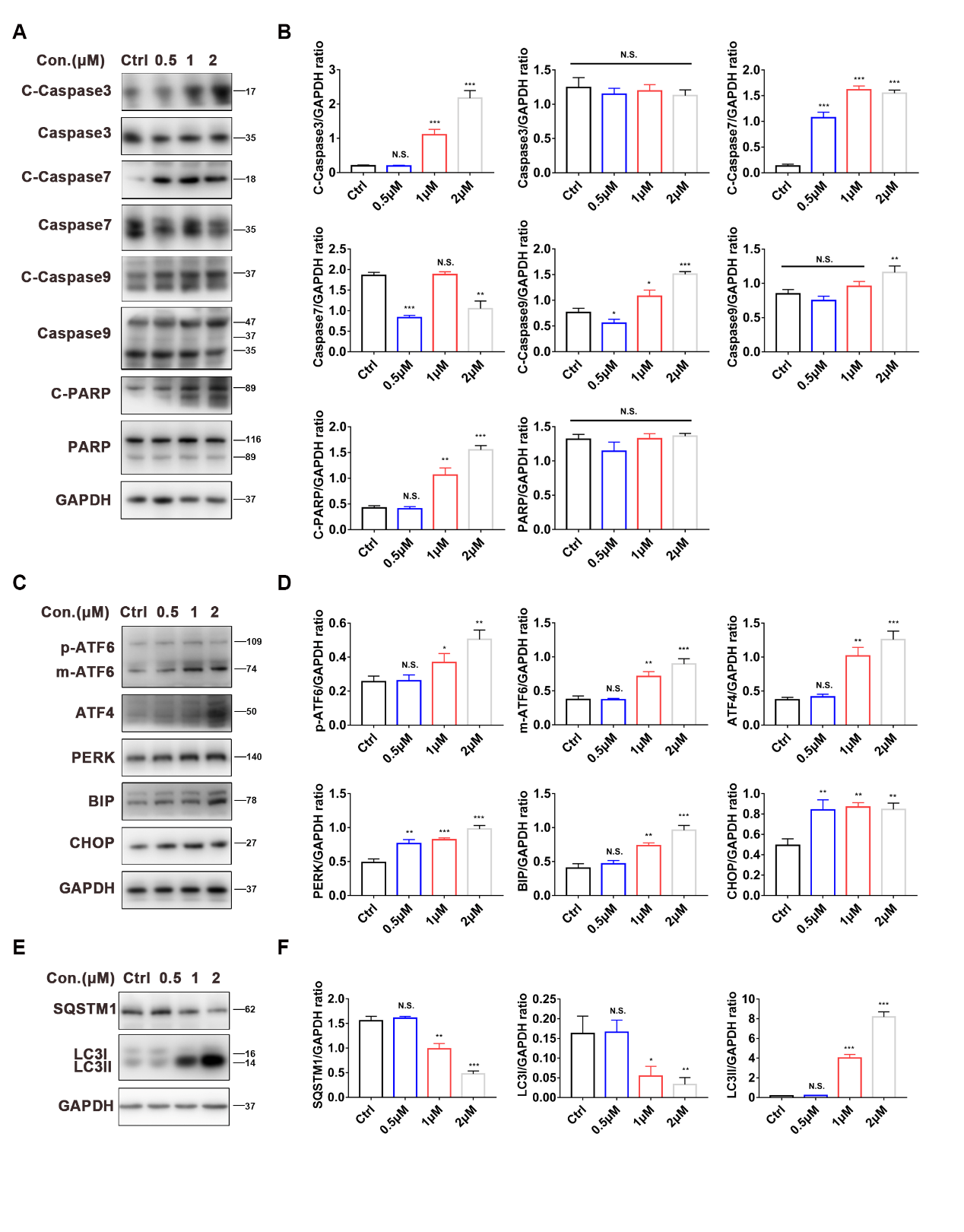


**Fig. S5 OTP-BTFPTU liposomes causes osteosarcoma cells death through multiple pathways in vitro. (A)** The expression of apoptosis proteins was measured by Western blot analysis in HOS cells treated with varying concentrations of OTP-BTFPTU liposomes for 24 h. **(B)** Quantification and normalization of the gray levels of C-Caspase3, Caspase3, C-Caspase7, Caspase7, C-Caspase9, Caspase9, C-PARP and PARP proteins to that of GAPDH in HOS cells using Image J. **(C)** The expression of ER stress proteins was measured by Western blot analysis in HOS cells treated with varying concentrations of OTP-BTFPTU liposomes for 24 h. **(D)** Quantification and normalization of the gray levels of p-ATF6, m-ATF6, ATF4, PERK, BIP and CHOP proteins to that of GAPDH in HOS cells using Image J. **(E)** The expression of autophagy-related proteins was measured by Western blot analysis in HOS cells treated with varying concentrations of OTP-BTFPTU liposomes for 24 h. **(F)** Quantification and normalization of the gray levels of SQSTM1, LC3I and LC3II proteins to that of GAPDH in HOS cells using Image J. The data represent the mean ± SD of three independent experiments. *p < 0.05, **p < 0.01, ***p < 0.001 for a comparison with the control group or as indicated.


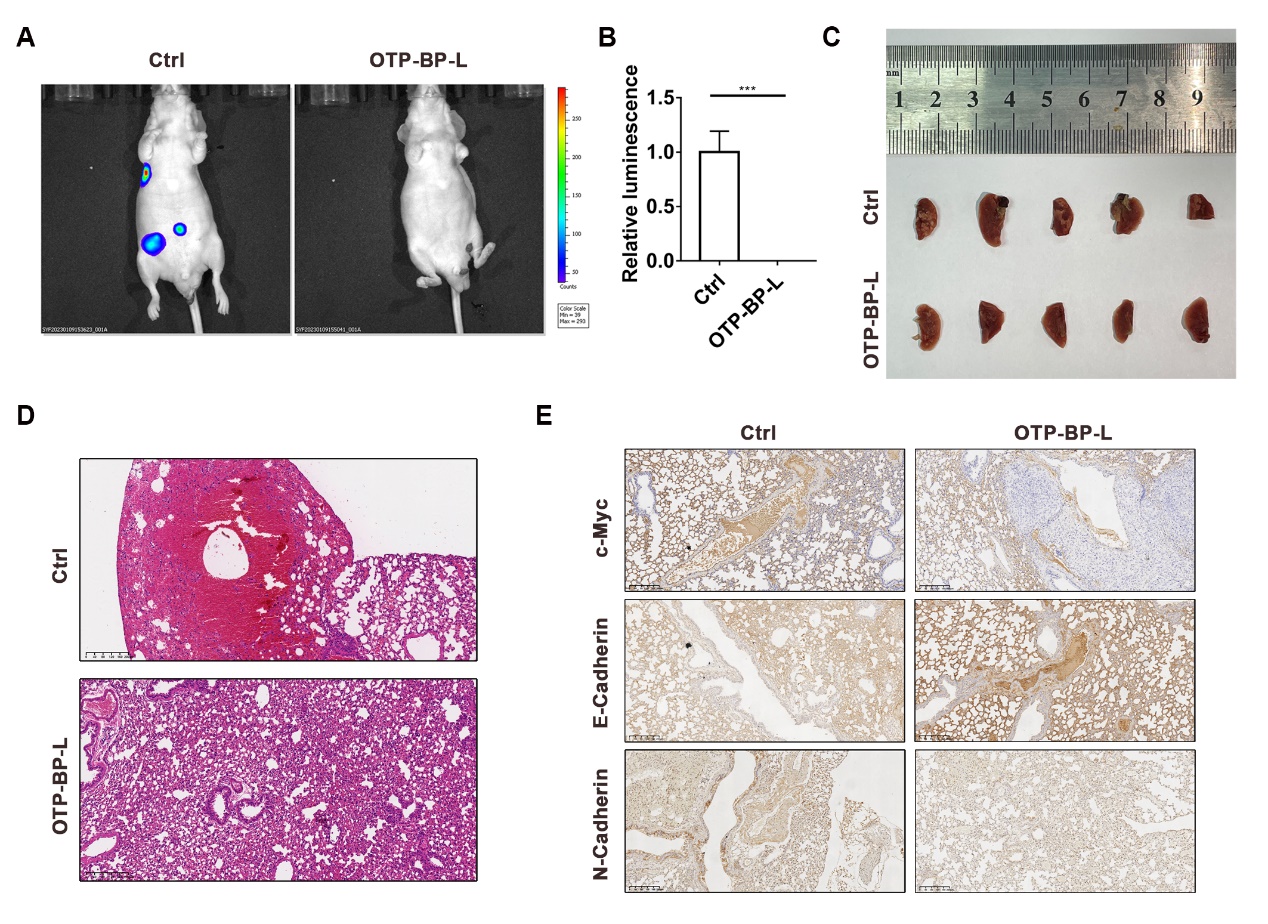


**Fig. S6 OTP-BTFPTU liposomes suppresses osteosarcoma tumorigenesis and metastasis in vivo. (A)** Living luminescence imaging of lung metastasis model mice with saline or OTP-BTFPTU liposomes treatment (n = 5). Representative images are shown. **(B)** Quantification of the relative luminescence of lung metastasis model mice with saline or OTP-BTFPTU liposomes treatment. **(C)** Photographs of HOS derived lung metastasis model with saline or OTP-BTFPTU liposomes treatment (n = 5). **(D)** HE staining of lungs which were obtained from different groups. Scale bars, 300 μm. **(E)** The expression of c-Myc, E-cadherin and N-cadherin were determined by immunohistochemistry. Scale bars, 200 μm. The data represent the mean ± SD of three independent experiments. *p < 0.05, **p < 0.01, ***p < 0.001 for a comparison with the control group or as indicated.


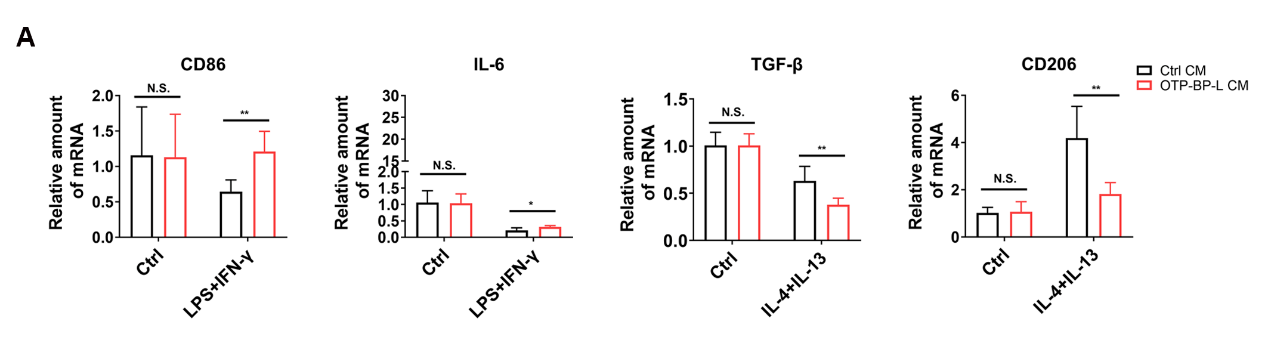


**Fig. S7 OTP-BTFPTU liposomes has the ability to regulate the tumor immune microenvironment. (A)** The mRNA level of CD86, IL-6, TGF-β and CD206 in BMMs following M1 or M2 macrophage induction in the presence of 50% control or OTP-BTFPTU liposomes CM. The data represent the mean ± SD of three independent experiments. *p < 0.05, **p < 0.01, ***p < 0.001 for a comparison with the control group or as indicated.
